# The knowns and unknowns of long COVID-19: from mechanisms to therapeutical approaches

**DOI:** 10.3389/fimmu.2024.1344086

**Published:** 2024-03-04

**Authors:** Roxana Gheorghita, Iuliana Soldanescu, Andrei Lobiuc, Olga Adriana Caliman Sturdza, Roxana Filip, Adela Constantinescu – Bercu, Mihai Dimian, Serghei Mangul, Mihai Covasa

**Affiliations:** ^1^ Victor Babes University of Medicine and Pharmacy, Timisoara, Romania; ^2^ Department of Biomedical Sciences, College of Medicine and Biological Science, University of Suceava, Suceava, Romania; ^3^ Integrated Center for Research, Development and Innovation for Advanced Materials, Nanotechnologies, Manufacturing and Control Distributed Systems (MANSiD), University of Suceava, Suceava, Romania; ^4^ Suceava Emergency Clinical County Hospital, Suceava, Romania; ^5^ Institute of Cardiovascular Science, Hemostasis Research Unit, University College London (UCL), London, United Kingdom; ^6^ Department of Computer, Electronics and Automation, University of Suceava, Suceava, Romania; ^7^ Department of Clinical Pharmacy, USC Alfred E. Mann School of Pharmacy and Pharmaceutical Sciences, University of Southern California, Los Angeles, CA, United States; ^8^ Department of Quantitative and Computational Biology, USC Dornsife College of Letters, Arts and Sciences, University of Southern California (USC), Los Angeles, CA, United States; ^9^ Department of Basic Medical Sciences, Western University of Health Sciences, College of Osteopathic Medicine, Pomona, CA, United States

**Keywords:** SARS-CoV-2, Inflammatory markers, brain fog, vascular injury, PASC

## Abstract

The coronavirus disease 2019 (COVID-19) pandemic caused by SARS-CoV-2 has been defined as the greatest global health and socioeconomic crisis of modern times. While most people recover after being infected with the virus, a significant proportion of them continue to experience health issues weeks, months and even years after acute infection with SARS-CoV-2. This persistence of clinical symptoms in infected individuals for at least three months after the onset of the disease or the emergence of new symptoms lasting more than two months, without any other explanation and alternative diagnosis have been named long COVID, long-haul COVID, post-COVID-19 conditions, chronic COVID, or post-acute sequelae of SARS-CoV-2 (PASC). Long COVID has been characterized as a constellation of symptoms and disorders that vary widely in their manifestations. Further, the mechanisms underlying long COVID are not fully understood, which hamper efficient treatment options. This review describes predictors and the most common symptoms related to long COVID’s effects on the central and peripheral nervous system and other organs and tissues. Furthermore, the transcriptional markers, molecular signaling pathways and risk factors for long COVID, such as sex, age, pre-existing condition, hospitalization during acute phase of COVID-19, vaccination, and lifestyle are presented. Finally, recommendations for patient rehabilitation and disease management, as well as alternative therapeutical approaches to long COVID sequelae are discussed. Understanding the complexity of this disease, its symptoms across multiple organ systems and overlapping pathologies and its possible mechanisms are paramount in developing diagnostic tools and treatments.

## 1 Introduction

COVID-19 pandemic has caused an unprecedented worldwide health and socioeconomic crisis leading to more than 768 million cases of viral infections, of which approximately 7 million deaths and 13,490,832,730 vaccine doses administered globally (1). The pandemic was caused by SARS-CoV-2, severe acute respiratory syndrome coronavirus-2 which infects the host by invading cells via ACE2–angiotensin-converting enzyme 2 (2). Although the respiratory tract is the site of entry and infection of SARS-CoV-2, COVID-19 is a complex disease, affecting the cardiovascular, renal, hematological, gastrointestinal, and central nervous systems, and can present a wide severity spectrum, from asymptomatic to severe, moderate or mild symptoms. The occurrence of acute COVID-19 last from 1-2 weeks in mild cases and up to 12 weeks for the most severe ones, based on factors such as age, symptoms, comorbidities, vaccination status, access to treatment and medical services (3). More than half of the infected individuals are presented with persistent symptoms even longer than four weeks after the onset of first clinical signs, a condition defined as post-acute sequelae of COVID-19 (PASC). The presence of clinical symptoms in infected individuals that continue at least three months after the onset of disease or with new symptoms that last for more than two months with no other explanation and that cannot be associated with other existing pathologies has been defined as long COVID-19 (L-C19) (4). The National Institute for Health and Care Excellence (NICE) classifies L-C19 in two categories: 1) “ongoing symptomatic C19” with symptoms that persist from 4 to 12 weeks and 2) “post C19” with persisting symptoms beyond 12 weeks after disease onset. Several other terms have been used based on the length and persistence of symptoms to define L-C19, such as “post-acute sequelae of SARS-CoV-2 infection”, “persistent C19 symptoms”, “post C19 syndrome” (PCS), “long haulers”, or “post C19 manifestations” (5, 6) (**Table 1**).

**Table 1:** Types of long-COVID.

Terminology	Persistence of symptoms after C-19
Long-COVID -19	> 8 weeks, without association with other existing pathologies; post-C19 with persistent symptoms > 12 weeks (NICE)
Long-term C19	> 4-12 weeks
Post-C19	> 8 weeks
Post-acute sequelae of COVID-19 (PASC)	>4 weeks after the first signs
Persistent post - C19	> 24 weeks
Long-haul C19	> 100 days

In L-C19, the virus is no longer present in the nasal cavity (7), however, viral protein and/or RNA has been detected in the reproductive and cardiovascular system, brain, muscles, eyes, olfactory mucosa, lymph nodes, appendix, breast, hepatic and lung tissue, plasma, intestinal microbiome, and urine (8, 9).

## 2 Long COVID-19 symptoms and predictors

**Figure 1:**
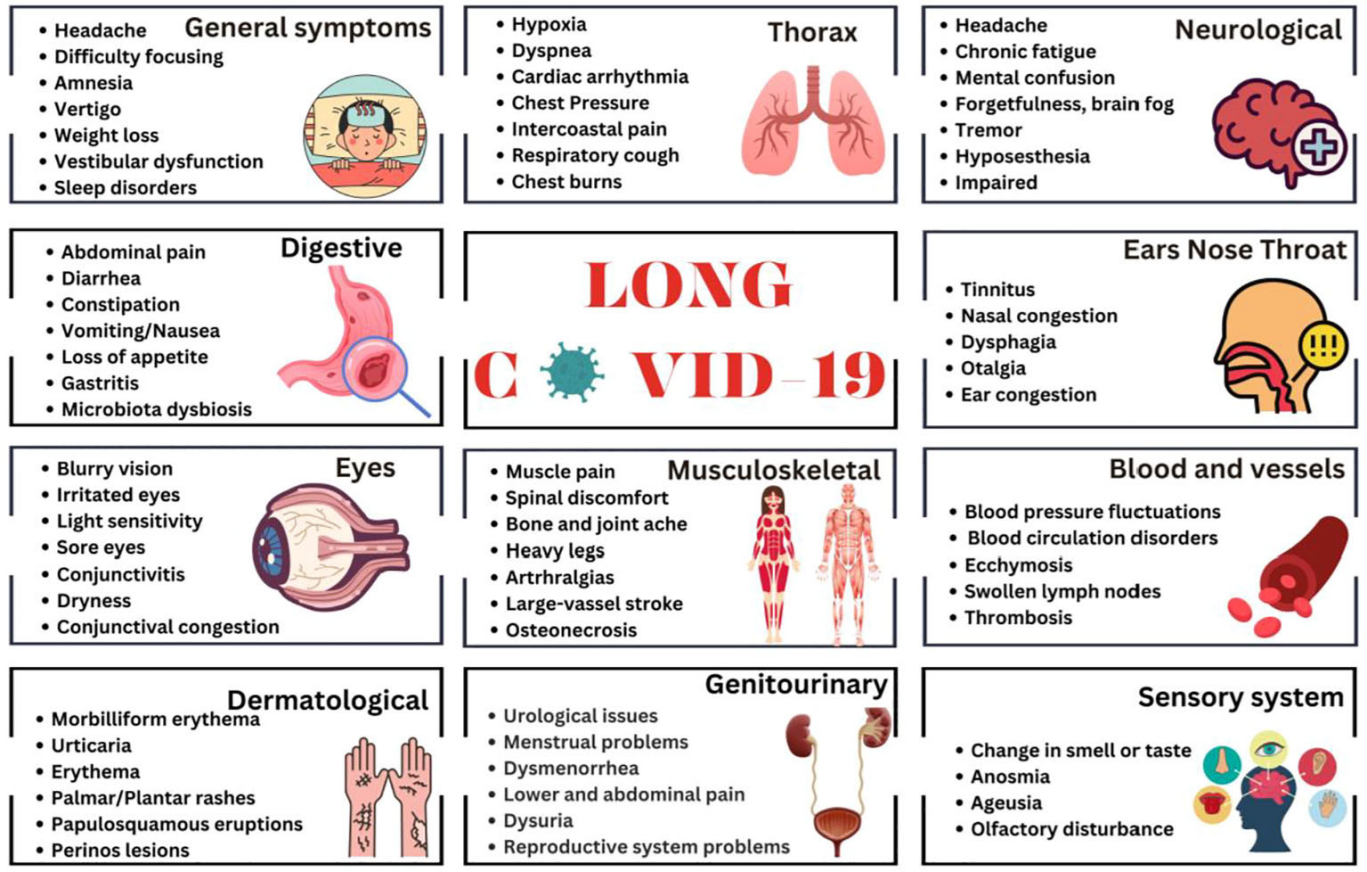
Commonly recognized symptoms associated with long COVID-19.

Given the wide spectrum of the L-C19 clinical symptomatology, establishing with certainty the syndrome, clinical manifestations, pathogenic factors, or its time framework, had proved challenging. The most common and representative symptoms of L-C19 include fatigue or muscle weakness, malaise, dyspnea, headache, dizziness or “brain fog”, depression, irritability, frustration, insomnia, and many other neurological disorders (10, 11). Other symptoms are related to cardiac, digestive, respiratory, reproduction, or dermatologic disorders. A recent meta-analysis study showed that the five most relevant physiological signs are fatigue, headache, deficit of attention, hair loss and dyspnea, followed by skin rashes, palpitations, and diarrhea (12), with recurrent spikes of fever as common symptom, but higher than observed after common infections, such as Epstein-Barr virus or influenza (13). The clinical presentation and symptomatology of L-C19 is similar with that of Chronic Fatigue Syndrome/Myalgic Encephalomyelitis (CFS/ME) (14), a known complicated illness with 4-6 months of fatigue and exhaustion, reduced daily activity and post-exertional malaise (15). Other common symptoms may include myalgia, muscle weakness, headache, sleep disorders, neurocognitive and psychiatric manifestations, anorexia or autonomic manifestations (orthostatic intolerance, cardiovascular, respiratory, gastro-intestinal, or gastro-urinary) (16) Compared with influenza, sequelae of L-C19 were higher in terms of anxiety and mood disorders, insomnia, and dementia (17). There have been over 200 symptoms associated with L-C19, with the most representative being depicted in **Figure 1**.

Due to the over-production of pro-inflammatory cytokines, C19 is not a usual viral pneumonia, but rather one with major consequences on the central and peripheral nervous system, cardiovascular, respiratory, urinary, immune system or on metabolic functions (11). For example, *Østergaard et al.* using scanning microscopy reported the presence of SARS-CoV-2 particles in the endothelium of lung, heart, kidney, brain or skin of C19 patients, with capillary changes and inflammation (18). It appears that L-C19 manifestation does not usually depend on the severity of the acute COVID-19 illness. In a 14-month study de *Miranda et al.* showed that most patients (75.4%) who experienced L-C19 had moderate infections and only 33.1% had been severe (19). Similar results were observed by *Sugyiama et al.* who reported that 49.5% from the patients identified with L-C19 were mild cases (2). Unfortunately, due to the multiple symptomatology and its undefined nature, it is difficult to detect L-C19 through laboratory findings. Thus, guidelines and regulations would be of great benefit in identifying L-C19. To this end, *Roth & Gadebusch-Bondio* proposed, in addition to conventional measures, presentation of cases, symptoms, and side effects through mass media platforms that are easily accessible globally (20). This could facilitate a more rapid self-identification of L-C19 symptoms, thus enhancing the possibility for treatment in a much shorter time.

Approximately half of the individuals infected at some point with SARS-CoV-2 developed L-C19. Exhaustion, cognitive dysfunction, myalgia, shortness of breath, chest pain or muscle aches were observed in most of these patients. Most of them confirmed that L-C19 affected self-care (50%), mental health (64%), and overall work (75%) (21). The greater risk of developing L-C19 was associated with the number of initial symptoms, with five or more being strongly correlated with persistent symptoms. In addition, hypertension was the most significant comorbidity associated with the development of L-C19, followed by diabetes, smoking, chronic cardiovascular or lung disease, and chronic kidney failure (22, 23). L-C19 has been more frequently encountered in individuals who have been reinfected with COVID-19 (24).

**Figure 2:**
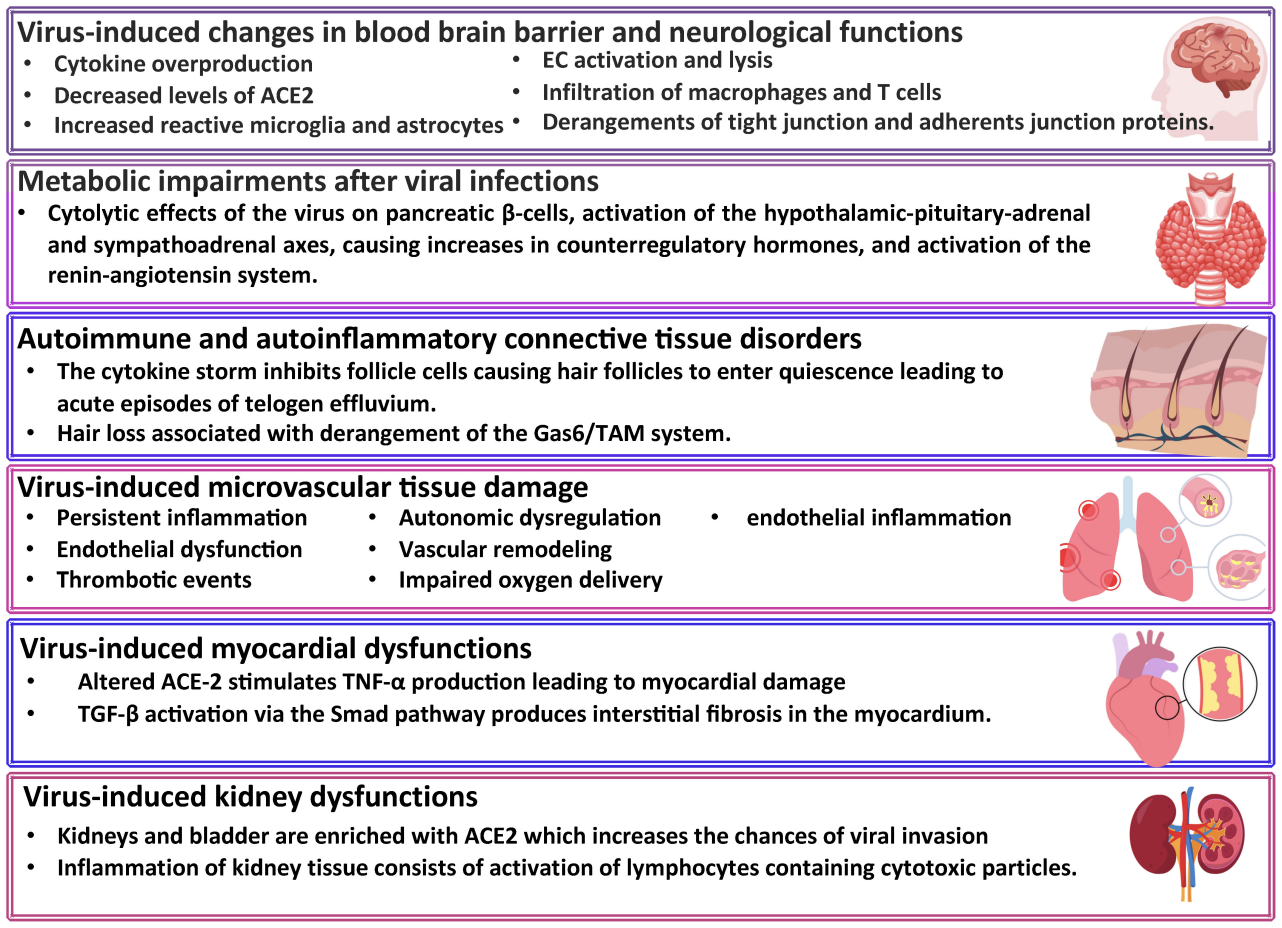
Long COVID-19 sequelae. ACE2, angiotensin converting enzyme-2; Gas6, gamma-carboxyglutamic acid protein; TAM, Transient abnormal myelopoiesis; TNF-α, tumor necrosis factor-alpha; TGF, transforming growth factor.

Clinical predictors of C19 involved increased levels of troponin, high white blood cell counts, blood sugar level, elevated cytokine response (tumor necrosis factor-alpha, interferon-gamma, C-C motif chemokine ligand 5, interleukin 6, IL-8, and IL-18) and altered gut microbiota. To establish L-C19 diagnosis, recommendations include electrocardiogram and transthoracic echocardiogram, as well as laboratory tests for CRP, troponin-T, pro-inflammatory markers (TNF-α, C-C motif chemokine ligand 5, IL-6, IL-8, IL-18, and interferon-gamma) levels. Moreover, several studies have identified a hypercoagulable state in L-C19 patients (25–27), with D-dimer levels being monitored (28) while other studies showed that reactivation of latent viruses or chronic inflammation led to L-C19. Thus, individuals with L-C19 symptoms presented chronic inflammatory and autoimmune conditions due to high level of monocytes and low levels of circulating cDC1, a type 1 dendritic cell with a role in immunity and viral infection (29). Several main mechanisms of L-C19 and its effects are presented in **Figure 2**.

## 3 Central nervous system clinical manifestations

### 3.1 Chronic fatigue

Multiple studies have shown that chronic fatigue is the underlying symptom in L-C19 (30–35). This led to significant interest in understanding the mechanisms and develop appropriate treatment strategies. It is known that chronic fatigue syndrome and depression are immune, oxidative and nitrosative stress-related disorders. These are significantly correlated with increased levels of inflammatory mediators such as C-reactive protein, increased aldehyde formation due to oxidative damage, increased nitric oxide production and hyper nityrosylation and low antioxidant levels (zinc, selenium, glutathione SH groups, glutathione peroxidase, total antioxidant capacity) (36). Several studies have suggested that certain vaccines such as those produced by Astra Zeneca and Pfizer may trigger the neuropsychiatric symptoms, especially somatic symptoms of Hamilton Depression and Anxiety. They concluded that these vaccines may be associated with some L-C19 symptoms such as anxiety, depression, fatigue, autoimmune response, and increased production of spike protein (36). Since fatigue is a general characteristic of L-C19, *Twomey et al.* suggested that measurement of fatigue level should be considered and a fatigue scale (such as FACIT-F) should be applied, especially because a validated treatment for L-C19 fatigue is not known and exercise therapy is generally not beneficial (37). L-C19 fatigue was strongly linked with comorbidities such as high blood pressure, high cholesterol, rheumatoid arthritis, diabetes, previous blood clots, cardiovascular diseases, auto immune disease. Elevated levels of anti-nuclear/extractable-nuclear antibodies are strongly correlated with higher level of TNF-α and are linked to fatigue in various diseases including chronic fatigue syndrome and rheumatoid arthritis (38). Negative psychological and social factors have also been associated with chronic fatigue (39, 40). To establish the true effects of L-C19’s chronic fatigue, it is thus necessary to identify and eliminate other common causes of chronic fatigue, such as anemia, hyperglycemia, thyroid disorders, dehydration or diabetes (41).

### 3.2 Brain fog, concentration, forgetfulness

Cognitive dysfunction is a main L-C19 symptom occurring in approximately 70% of patients (42). The most representative symptoms are concentration difficulties, brain fog, forgetfulness, semantic disfluency, or tip-of-the-tongue (ToT) word-finding problems. According to *Guo et al.*, chronic fatigue and neurological symptoms observed in the first three weeks of illness can be a predictive factor for cognitive disorders (43). Most of these clinical manifestations are identified in patients who experienced severe C-19 illness and were hospitalized (42). Although brain fog has been reported after ordinary upper respiratory tract infections, its prevalence is significantly higher after C19, with 17.8% prevalence 2 months following acute C19 (44, 45). Using Montreal Cognitive Assessment (MoCA) score, *Alemano et al.* showed that more than 80% of patients with severe C-19 developed cognitive deficits later (memory, executive function, and language). It is interesting that patients who underwent sedation and ventilation presented fewer cognitive disorders, an effect attributed to reduction of stress by sedation and/or increased oxygenation due to ventilation (46). Several mechanisms have been proposed in these neurological disorders that include neuroinflammation, aberrant immune response between SARS-CoV-2 and body antigens, residual virus particle, metabolic brain disorders, and activation of peripheral trigeminal nerve roots (47). Other factors are structural or volumetric vascularization disorders, with high blood pressure or high cholesterol. Unfortunately, these comorbidities and C19’s cognitive disorders are strongly associated with loss of grey matter within the temporal lobe which, along with reduced memory performance, may be an increased risk for neurodegeneration or dementia (48). This hypothesis was confirmed by *Hugon et al.* who observed abnormal hypometabolic area after cerebral FDG PET scans of L-C19’ patients. Thus, the abnormal neurological functions may be attributed to deficient brain connections between the anterior and posterior cingulate cortex. The cingulate cortex is involved in memory, emotions, depression, or action decision, and its hypometabolism have been observed in neurological and psychiatric diseases, such as Alzheimer, depression or internet gaming disorder (49). The neurocognitive and neuropsychiatric deficits change CNS immune and glial cells and the negative effect on neuronal pathophysiology is mainly due to myelin homeostasis and plasticity disturbance, hippocampal neurogenesis impairment with neurotoxic astrocyte reactivity (50). The differences in white matter were observed only three months after acute phase and completely recovered after 10 months. After 3 months, a self-recovery mechanism of neuroplasticity was observed (51). These effects were mainly observed in females, patients with respiratory problems or those who needed ICU interventions (52).

Some of the medications received in C-19 acute infection may also affect the neurological system of patients as well. Thus, the neurological side effects of drugs such as lopinavir-ritonavir and corticosteroids should be considered when assessing L-C19 effects (28). More recent studies suggested that brain fog in L-C19 patients may be caused by the presence of blood clots in the cerebral or pulmonary circulation (53) and were associated with increased serum ferritin levels during C19 hospitalization (45). Since cognitive disorders are difficult to manage and treat, many patients with cognitive impairments, require long-term psychological support and treatment. Unfortunately, cognitive dysfunction led to reduced work capacity, prolonged time of normal activities, difficult work ability, mental exertion, stress and loss of employment, thus having a severe impact on the quality of life (54).

### 3.3 Headache

It is one of the earliest and most common symptoms of L-C19 and it is usually accompanied by hyposmia, fatigue, dyspnea, myalgia, and cough. It is influenced by C-19 acute phase severity and the use of analgesics, genetic predisposition to migraine due to trigeminovascular system activation, and systemic immune response at viral infection (55). Without a specific clinical phenotype, the headache topography is predominantly bilateral, with frontal or periocular presence, and the pain is felt like a tension-type headache, usually without any symptoms, or migraine-type headache, accompanied by vomiting or nausea (56). The presence of headache in C-19 acute phase have been considered a positive prognosis, being associated with lower severity, lower mortality, and lower need of ICU interventions (57). Some symptoms, such as headaches, continue to persist to a significant extent in the long term. For example, 16.5% of patients continue to experience headaches after 60 days from the start of the disease, of which 8.4% reported significant headaches even after 180 days post COVID-19 (58, 59). Thus, symptoms such as headaches can persist to a significant extent even months after the onset of the disease, highlighting the importance of a holistic approach and continued research to better understand the long-term health impact of this disease.

### 3.4 Depression and anxiety

Depression and anxiety are found in over 50% of patients with L-C19, especially due to hospitalization or cognitive disorders (60). Even if these symptoms were not identified in the first phase, they are usually found in patients with certain difficulties, being part of the long-term symptoms category. The first psychiatric sequelae were noticed 14-90 days after C-19 and the estimated probability to develop new sequelae after 90 days was 5-8%. The anxiety disorders are shown to be more prevalent than mood disorders (53). Some authors suggested that depression and anxiety may be a result of disease severity and trauma of hospitalization rather than of the viral infection (61). Due to individuality of each patient, presentation can vary which makes the treatment challenging (62) thus early diagnosis and close follow-up is critical. Unfortunately, depression and anxiety are difficult to identify since they are intrinsically related to patients’ overall health and life quality. When comparing COVID infected patients with or without symptoms of depression and anxiety, imaging tests via MRI showed atrophy of the brain areas responsible for processing emotions and memory. Thus, it is clear that the anxiety and depression caused by this virus have significant long-term consequences (63).

### 3.5 Sleep disorders

Sleep disorders and fatigue are the most persistent symptoms that affect the daily life of L-C19 patients (64). More than 40% of patients experienced insomnia, however, the symptoms gradually improved after 3 months. When tested with wearable health devices, L-C19 patients reported decreased total sleep and light or deep sleep time leading to related health issues (65). In some patients, sleep disturbances were present even after 12-month post C-19, with negative impact on patients’ quality of life. This, along with fatigue, altered respiratory functions, stomach burn, or abdominal pain add to the significant burden of L-C19 patients (66, 67).

### 3.6 Cognitive disorders

Most patients seem to return to normal life after infection with C-19, but many remain with mild symptoms that are often attributed to other daily factors such as fatigue, memory problems, and loss of attention, without realizing that these symptoms may be long-term symptoms caused by C-19 infection (68). Research shows that 1 in 8 patients may receive a neurological diagnosis after C-19, even after 6 months post SARS-2 infection (69). Several biomarkers have been used to differentiate between LC-19 and other causes of cognitive disorders symptomatology; however, research thus far is relatively scarce. One promising study analyzed fibrinogen and D-dimer compared to C-Reactive Protein (CRP) levels in more than 1830 hospitalized patients and found positive correlations between cognitive impairment and COVID-19 infection (70). Subjective as well as objective cognitive deficits were analyzed, considering occupational effects. Using canonical correlation (CCA) methodology to assess associations between sets of variables, it was shown that increased fibrinogen was correlated with both objective as well as subjective cognitive deficits at 6 and 12 post COVID (70). Fibrinogen has a direct effect on blood clotting, but it also fulfils site-binding functions that could affect axon binding or even have effects that degrade the β-amyloid protein (71, 72). High levels of D-dimer were associated with subjective cognitive difficulties and occupational effects (69). An increased level of D-dimer has been associated with a risk of pulmonary thromboembolism, which may be related to poor oxygenation of the brain, with direct implications for cognitive impairment (73). Determination of cognitive impairments after C-19 infection is a complex process and requires identification of factors and mechanisms of action of different biomarkers, including correlation with brain imaging.

## 4 Peripheral nervous system and other organs clinical manifestations

### 4.1 Anosmia/ageusia or altered smell and taste

Smell and taste dysfunctions are the most prevalent symptoms in L-C19, after fatigue (74, 75). Anosmia was more frequent present in patients with mild C-19 forms and less reported in patients with severe C-19, eye, nose and throat complaints. Initial studies demonstrated that, in mild C-19 cases, patients might have had stronger local immunity and the virus replicated in the mucosa (76). Persistent olfactory dysfunction has been associated with poorer emotion recognition in patients who experienced mild C-19 (77). The persistence of anosmia or ageusia in L-C19 patients has been attributed to injuries in the olfactory neuronal pathways or persistence of neuroepithelium inflammation (47). However, in patients with previous mild SARS-CoV-19 infection, the 3-year prevalence and recovery rate of COVID-19-related alteration in sense of smell or taste was 5% and 92%, respectively. In patients experiencing chemosensory dysfunction still 2 years after COVID-19, a delayed complete or partial recovery has been observed even after 3 years, while some patients continue to have unchanged persistent chemosensory alteration (78). Gene expression profiling of olfactory epithelium of patients with persistent olfactory symptoms showed changes in the expression levels of miRNAs involved in neural development and immune response. Further, overexpression of metallothioneins, in response to increased inflammation of the olfactory epithelium may results in decreased zinc levels and subsequent loss of smell and taste (79). L-C19 chemosensory disfunction significantly reduces quality of life, with negative implications in mental health: anxiety, depression, loss of appetite, weight changes, even work and study difficulties or social and interpersonal limitations (80).

### 4.2 Renal system clinical manifestations

Among LC-19 sequelae related to the renal system were acute kidney injury or renal failure, mainly due to high abundance of pf ACE2 expression in kidneys, with declined glomerular filtration rate or kidney infarction, mainly due to thromboembolism. The prognosis is associated with significant risks of mortality or morbidity. Acute kidney injury was observed mainly in non-survivors and in 1% of survivors, with renal function restauration. Therefore, investigation tools should include early recognition of kidney functional impairments or injury through urine analysis, glomerular filtration rate, ultrasound scanning or renal biopsy (81–83).

### 4.3 Gastrointestinal clinical manifestations

The main gastrointestinal (GI) symptoms shown to persist even six months after C-19 acute phase are constipation, diarrhea, abdominal pain, nausea/vomiting, and heartburn. Patients who reported L-C19 anxiety or sadness were more prone to present GI symptoms (55% vs. 14%) and conversely, GI symptoms lead to anxiety/sadness (84). The possible mechanisms involve the abundance in ACE2 and furin expression, fecal-oral transmission, lymphocytic infiltrations into intestinal tissues’ lamina propria, intestinal dysbiosis, or high cytokine levels (81). Inflammation and intestinal metabolites dysfunction are influenced by nutrition, diet, malnutrition, old age or diabetes and obesity (85). Further, gut microbiota dysbiosis, GI peripheral tissue damage and altered immune status have been reported six months after the infection (86). The investigation tools should include colorectal transit observations, CT scan, defecography and swallowing studies (81).

### 4.4 Cardiovascular and respiratory clinical manifestations

Approximately half of patients with L-C19 reported incomplete recovery, with shortness of breath and other respiratory problems (37, 87), that included chest pain, cough, or sputum production (88). The breathing discomfort has been attributed to chronic changes in breathing pattern and persisting inflammation in lungs or mediastinal lymph nodes, as well as association of metabolic abnormalities with lung sequelae. These pulmonary vascular network abnormalities have been the cause for pulmonary hypertension and damaged respiratory reflexes due to intrathoracic receptors destruction (47). L-C19 patients developed endothelial dysfunction which, even if it had progressively improved after 6 months, the risk for thrombotic or cardiovascular events remained high. Usually, endothelial dysfunctions develop in the acute phase of the disease and were responsible for atherosclerosis. These symptoms persisted even 6-months after C-19 in 58% of patients. There was a negative correlation between IL-6 levels and brachial artery flow-mediated dilation, an effect that was improved by treatment with tocilizumab, an IL-6 inhibitor. Patients hospitalized in a medical ward recovered faster than those hospitalized in ICU (89, 90). The presence of microclots associated with insoluble inflammatory molecules, antibodies and immunoglobulins were present not only in the acute phase of C-19, but also played a critical role in the development of L-C19 symptoms and other auto-immune pathologies. For example, increased level of galectin-3-binding protein (responsible for cancer development, progression and metastasis) inflammatory markers (IFN-α, IFN-β, IFN-γ, TNF-α), trombospondin-1(increased in tumors and associated with thrombosis), α-1-acid glycoprotein-2 (associated with demyelinating diseases), and reduced levels of long palate, lung and nasal epithelium carcinoma-associated protein 1, lactotransferrin, adiponectin and α-1-acid glycoprotein-1 may be the result of immunosuppression similar to that of sepsis. Evidence suggested that if these microclots resolved in the first phase of the disease, hypoxia could be avoided. This is an important finding, especially because hypoxia led to irreversible tissue damage and can be life threatening for patients with diabetes 2 or cardiovascular co-morbidities (91). For example, fibrin amyloid microclots blocked capillaries and impeded O2 to be transported to the tissues, leading to thrombotic events, myocardial infarction, strokes, kidney disfunction, or neurological disorders. Thus, their removal could be a potential therapeutic strategy which may allow the body the possibility to self-repair (92, 93). The presence of ‘amyloid’ microclots and platelet hyperactivation has prompted some research groups to propose a triple anticoagulant therapy as a potential treatment of L-C19, though this needs to be further assessed (27, 92). Furthermore, studies showed an increase in thrombin generation in patients with L-C19 (26, 94), in line with the presence of a hypercoagulable state in these patients. Persistently raised D-dimers and sustained inflammation has also been identified in convalescing C-19 patients who were not hospitalized during acute infection (24, 95, 96). More recently, a study using microfluidic strategies to reflect the physiological conditions of the vasculature, revealed that patients suffering from L-C19 have higher thrombogenicity under flow, with increased platelet capture and larger thrombi compared to matched healthy controls. These patients had been suffering of L-C19 for an average of 23 months, suggesting that this emerging disease might cause long-term thrombogenicity. These findings were correlated with levels of Von Willebrand Factor (VWF) or ADAMTS13 activity, involved in arterial thrombotic pathologies (26). An increased VWF concentrations and decreased ADAMTS13 activity were correlated with a higher risk for myocardial infections (97). Indeed, patients with L-C19 were also found to have an increased VWF : ADAMTS13 ratio, above 1.5, which was associated with impaired exercise capacity (98, 99).

Comorbidities such as high blood pressure, high cholesterol, rheumatoid arthritis, or cardiovascular diseases can lead to respiratory system symptoms. NICE recommends that breathlessness should be auto identified through simple tolerance test exercises and blood oxygen levels with pulse oximeter. For example, Mayo Clinic suggests that smoking, pollutants and extreme temperature may contribute to L-C19 pulmonary symptoms’ persistence (39). WHO rehabilitation guidelines include precautions regarding gradual return of daily activities of L-C19 patients and recommends that physical exercise must be adapted in order to prevent fatigue and patients’ activity must be in accordance with their symptoms (98). Other L-C19 symptoms are presented in **Table 2**.

**Table 2:** Additional long COVID-19 symptoms and mechanisms.

Disorder	Manifestation/Symptoms	Factors/Mechanisms	References
Corneal nerve fiber loss	Neurological	Microglial activation, lymphoid inflammation	(100)
Vascular endothelial damage	Coagulation, micro thrombosis, organ damage	Long-term viral infection hypoxia, inflammatory response	(101)
Epstein-Barr Virus Reactivation (the occurrence in the acute phase of the C-19 hospitalized patients predicts L-C19 symptoms)	Mononucleosis – associated symptoms and complications: fatigue, insomnia, headache, myalgia, confusion, brain fog, rash, urticaria, folliculitis, pharyngitis, abdominal pain, tinnitus, neck lymphadenopathy, mild hearing loss, cryoglobulinemia; myocarditis, inflammatory cardiopathy, myocardial infarctions; liver, kidney, and respiratory failure	Immune system weakness, physiological stressors, corticosteroid, and glucocorticoids treatments	(29, 102–105)
Mastocytosis	Allergic reactions	Histamine, IL-1 increased levels, promote and intensify the cytokine storm and inflammation	(106)
Motor peripheral neuropathy	Muscle weakness, atrophy, Achille reflexes absence, reduced/abnormal distal pin and vibration sensations	Intense inflammation, nerve compression, neuritis with perivascular macrophage infiltrates	(107)
Rheumatologic/ musculoskeletal	Joint pain, muscle pain, low back pain, back and neck pain, fatigue, myalgia	Cellular invasion, inflammatory and immune response, transformed growth factor beta (TGF-β) – persistent immunosuppression and fibrosis	(108)
Postural Orthostatic Tachycardia Syndrome	Fatigue, heart rate variability disfunction, orthostatic hypotension	Hypovolemia, neurotropism, inflammation and autoimmunity	(109)
Cancer development or progression	Immunosuppression, death	Cellular transformation, tumor suppressor protein p53 degradation, genomic instability, aberrant cell growth	(110)
Dermatological issues	Pernios lesions and livedo reticularis > 150 days; urticarial, erythema, purpura, palmar/plantar rashes	Microvascular changes	(111, 112)
	Alopecia	Inflammation, emotional distress	(113)
Metabolic disorder	Diabetes	Insulin resistance, abnormalities in glucometabolic control and β-cell function	(114–117)
Emotional	Confusion, fear, loss of individuality, insomnia, hopeless, loss of appetite, bad eating habits	Uncertainty, concerns regarding the incomplete recovery, misdiagnosed symptoms, cognitive deficits, anosmia, parosmia	(118–120)

## 5 Transcriptional markers for long COVID-19

In addition to clinical symptoms and alteration of biochemical markers’ levels, long COVID affects several regulatory processes in human body, in eluding changes at the transcriptional level. This comes from the fact that, the virus hijacks the host cell transcriptional/translational machinery during acute infection to produce large amounts of viral proteins and RNA, while shutting down host messenger RNA translation (8). For instance, persistent alterations in the blood transcriptome 446 genes displayed significant differential expression in individuals referred to a long COVID clinic compared to those not referred, at 24 weeks post-infection. No such differences were observed at earlier timepoints, suggesting that while many individuals note resolution in transcriptional dysregulation approximately 6 months post-infection, this is not the case for those with long COVID symptoms (121). Furthermore, pathway analysis of patient groups revealed four transcriptome groups. First, enriched with Th1-like signatures in CD4+ T cells, M1-like pro-inflammatory signatures in monocytes, cytotoxic effector signatures in CD8+, T cells and NK cells, and memory signatures in B cells. Second, enriched for Th2-like CD4+ T cell signatures, M2-like (anti-inflammatory) monocyte signatures, and a plasma B cell signature. Third, a transitional immune status between types 1 and type 2. Fourth, a naive group, with naive-like T and B cell signatures, and resting NK cell signatures (122).

Transcriptional modifications may lead to disease progression, as transcriptomic analysis of bronchoalveolar lavage from severe COVID-19 patients showed reduced IFN-responsive genes response, compared to blood. Also, downregulation of interferon stimulated genes such as MX1, IFITM1, and IFIT2 were observed in critical COVID-19 cases, as well as undetected messenger RNA levels of IFN-β in blood (123). Nevertheless, transcriptional modifications may also lead to delays in recovery, as ongoing perturbations on a transcriptional level were observed for up to 6 months after infection, with PASC patients showing a distinct profile, such as upregulation of the alarmins S100A8 and HMGB1, mediators and markers of innate immune activation (124). Also, increased expression of acute inflammatory markers was observed 9 weeks after acute phase, also positively correlated with COVID-19 severity (125).

SARS-CoV-2-induced transcriptional modifications may be involved in inflammation and, possibly, processes like thrombosis. For example, transcriptomic analysis of platelets from SARS-CoV-2 patients revealed increased transcription of IL-6, tumor necrosis factor TNF-α, blood coagulation, and hemostasis, possibly leading to thrombosis (123). This behavior could suggest mild thrombocytopenia, which usually implies fatigue, that is common in long COVID, but also increases in S100B, a marker of neurological damage, which is linked to the neurological symptoms observed in long COVID (126). Tregs (regulatory T cells), a subset of CD4+ T cells displayed elevated transcriptional signatures inducing correspondent high levels of proinflammatory molecules, which may lead to reduced antiviral T cell responses in acute COVID-19, while also promoting inflammation (127).

The role of Tregs in the context of Long COVID, particularly regarding transcriptional markers, represents an area of active investigation with emerging insights. Tregs, known for their immunosuppressive and immunoregulatory properties, play a significant role in the prognosis of COVID-19. Patients with COVID-19 are reported to have fewer Tregs compared to the general population, leading to diminished inflammatory inhibition and an increased likelihood of respiratory failure and long COVID development (128). Further, Tregs exert control over both adaptive and innate immune responses through various mechanisms. They produce cytokines such as TGF-beta, IL-10, and IL-35, which inhibit T cells, leading to suppressed actions of Th1, Th2, and Th17 type T cells. Tregs also directly affect B cells and inhibit macrophages, further highlighting their comprehensive role in modulating the immune response. An imbalance in Treg numbers can have deleterious effects by limiting antiviral effects of effector T cells and contributing to an excessively stimulated immune response in severely infected patients (128).

In a longitudinal analysis, immunological, inflammatory, and metabolic data were collected from patients to generate a composite signature predictive of systemic recovery. The study found intrapatient covariation of innate immune cell numbers, levels of kynurenine and lipid metabolites, and other molecular and cellular parameters that predicted recovery, mortality, and post-acute sequelae of SARS-CoV-2 infection. Notably, patients with persisting inflammation, a characteristic of the recovery group in this study, had reduced Treg cell counts (129).

A further longitudinal transcriptome analysis, which emphasized robust T cell immunity during recovery from COVID-19 showed that, during recovery, there was a significant downregulation of humoral immunity and type I interferon response, alongside upregulation in genes involved in T cell activation and differentiation. These findings support the role of T cell immunity, including the function of Tregs, in the immune protection against COVID-19 (130). Also, a scoping review that focused on Treg dynamics in convalescent COVID-19 patients showed that while Treg populations can reconstitute during recovery, there is an observed dysregulation in the Treg compartment that can persist for months. This dysregulation may be linked to the immune system-associated sequelae observed in Long COVID patients (131). Therefore, the evidence suggests that Treg dysregulation, as reflected in altered transcriptional markers, plays a crucial role in the pathophysiology of Long COVID. These findings underscore the importance of further research to elucidate the complex interplay between Tregs and other immune components in Long COVID, which could potentially inform targeted therapeutic strategies.

The importance of transcriptome analysis for PASC assessment is underlined by the fact that a transcriptome-wide investigation showed that processes leading to PASC already start during hospitalization for acute COVID-19 and divergent etiologies for different sets of symptoms were identified, depending on the antibody response to SARS-CoV-2 spike protein. This points to the fact that study designs capturing only the post-acute phase may not take into account valuable explanations for pathogenesis of PASC (132). Furthermore, variable recovery rates in the transcriptome of COVID-19 convalescents were observed, as some convalescents return to baseline transcriptome within 24 weeks after infection, but not those with long COVID, suggesting persistent transcriptional dysregulation. This persistence of dysregulation might explain continued symptoms like fatigue post COVID-19 (126).

## 6 Risk factors for long COVID-19

At the onset of SARS-CoV-2 pandemic, heath care providers were caught off guard and, racing against time to identify solutions to limit the spread of the virus and treat C-19. Early studies identified several risk factors that influence the severity of C-19 such as age, gender, co-morbidities, persistent lesions, time of hospitalization, treatment, or lifestyle. When the spread was contained and more effective treatments and vaccines became available, the heath care communities were faced with another challenge which was that over half of the population that had C-19 showed symptoms of L-C19. Due to the long period of manifestation, this led to the exacerbation of existing diseases, triggering of new ones, but also to major changes in the quality of life. Thus, the recognition, diagnosis, and management of L-C19 became critical. Similar to the acute phase of C-19, L-C19 also depends on a series of risk factors such as sex, age, co-morbidities, severity of C-19, hospitalization and its duration, C-19 sequelae, the treatment applied in the acute phase, vaccination, as well as lifestyle.

### 6.1 Sex

All studies indicate that regardless of the severity of C-19, women are more likely to develop L-C19. Although the precise mechanisms for sex differences are not completely elucidated, the faster immune response of women, which protect them from initial infection and severity might be one important factor. Unfortunately, this makes females more vulnerable to prolonged autoimmune related diseases. Other sex differences include different sex hormones, high exposure and high psychological stress occupation. Furthermore, women have been shown to be diagnosed later than men (133) and are more likely to develop L-19 cognitive disorders, anosmia, dysgeusia, respiratory and rheumatological sequelae (134–137). On the other hand, men are more exposed to renal sequelae and endothelium disfunction (138–140). Other findings suggest that women had lower mortality and lower levels of inflammation than men (141) which may be due to the immunomodulatory effects of estradiol in females and higher antiplatelet and vasodilatory activity (142, 143). Conversely, men have higher risk of inflammation or tissue damage due to higher amount of cytokines and chemokines like IL-2, TNFα, IL-7, IL10, IL-18, CCL14, or CCL23 (144). Furthermore, it has been shown that production of IL-6 inflammatory marker is lower after viral infection in women which has been associated with a higher risk of developing L-C19 symptoms (145). In a meta-analysis study, *Notarte et al.* showed that females presented a higher risk of developing L-C19 symptoms such as dyspnea, fatigue, breathlessness, chest pain, palpitations, depression, sleep disorders, hair loss, ocular problems, and GI-related problems than men (146).

In pregnant women, C19 had more serious consequences in the acute phase, with premature births and even deaths among both mothers and newborns. In terms of the long-term effects of the virus on pregnant women, research shows a similarity between the symptoms of the general population and pregnant women (147, 148). For example, in their study conducted on 99 pregnant women, *Zhou et al.* demonstrated that 74.75% exhibited at least one symptom of L-C19 (presumably referring to a condition related to COVID-19), with the most common symptoms being fatigue, myalgia, and anosmia/ageusia (148). The same symptoms, to which difficulty in concentration and hair loss were added, have also been described by *Vásconez-González et al.* Most symptoms were initiated with infection (42.4%) and 3-5 weeks after infection (35.5%), which lasted between 3 and 6 months (21.2%), more than two months (18.6%) or between 4 and 8 weeks (17.8%) (149). Antenatal depression (25.2%) and antenatal anxiety (27.9%) were the most common symptoms observed in post-epidemic period among pregnant women. The prevalence of these symptoms were lower than those during pandemic, but higher than those from pre COVID period. The factors that most influenced the physiological state were psychiatric treatment history, psychological counseling before pregnancy, age, education, access to information, financial instability, trimester, pregnancy complications, number of hospital stays and poor marital relationship. Financial security and high levels of social support have led to a reduction in anxiety and depression among pregnant women (150–152).

### 6.2 Age

Although C-19 is less common in children than adults, L-C19 and multisystem inflammatory syndrome (MIS-C) are long-term consequences observed in asymptomatic patients (153, 154). Like adults, adolescent girls are also more prone to L-C19 than boys (155, 156). The main L-C19 symptoms reported in children and adolescents were neuropsychiatric: mood, fatigue, sleep disorder, headache, cognition, dizziness, neurological abnormalities (pins, tremor), balance problems; cardiorespiratory: respiratory symptoms, sputum/nasal congestion. orthostatic intolerance, exercise intolerance, chest pain, rhinorrhea, cough, sore throat, chest tightness, variation in heart rate, palpitations; dermatologic/teguments: hyperhidrosis, dermatologic (dry skin, rashes, hives), hair loss; gastrointestinal: abdominal pain, constipation, diarrhea, vomiting/nausea; and other: loss of appetite, altered smell, body weight variations, myalgia, altered smell, otalgia, ophthalmologic (conjunctivitis, dry eyes), fever, changes in menstruation, urinary symptoms, dysphagia, speech disturbances (157–159).

The quality of life of children and adolescents was not as impacted by L-C19 as that of adults. A small proportion reported feeling scared and worried and experienced a lack of friendship. School absence due to ill period affected their general wellbeing and it was a disturbance factor for both children and parents (160, 161). *Buonsenso et al.* reported that L-C19 greatly influenced the quality of life of children that included energy level (83.3%), mood (58.8%), sleep (56.3%), appetite (49.6%) and lack of concentration (60.6%). The authors concluded that most children had worse activity level than before infection and almost half of them experienced recovery from L-C19 symptoms’ cycles with 25% of them presenting constant symptoms) (162). The treatment strategy for these children should be multidisciplinary and the conventional approaches should be accompanied by changes in dietary habits (163). It is recommended that every affected child to be monitored by a primary care pediatrician four months after C-19 acute phase to check the presence of symptoms and development of new ones. The presence of L-C19 symptoms must be immediately considered and in absence of any doubt, the visit should be rescheduled after three months (164). Children with neuropsychiatric disorders must be followed up by mental health experts, pediatricians and must be supported by family and friends (165). An important issue that should be addressed is that during the pandemic, many children without C-19 experienced similar L-C19 symptoms with those infected: headaches, fatigue, sleep disturbance, and concentration difficulties. Several studies showed that almost all symptoms reported by SARS-CoV-2 infected children were also present in patients with negative test (145, 166).

### 6.3 Pre-existing conditions

L-C19 symptoms are more prevalent in individuals with underlying diseases, such as diabetes, obesity, cardiovascular or neuropsychiatric related disorders (167). Functional impairments of one or more organs due to C-19 acute phase was also strongly associated with L-C19 symptoms. Thus, coagulation issues, reactivation of some existing viruses (i.e., herpesviruses), dysfunctional nerve signaling, or autoimmunity are considered potential contributors of C-19 sequelae (168, 169). Asthma (39, 170), hyperthyroidism (171), hyperglycemia and dyslipidemia (172) have all been considered high risks for L-C19 development at old age patients. These pathologies degrade ACE2, leading to increase activation of the ACE2 receptors, virus entry and persistence of inflammatory cytokine storm or oxidative stress (145).

### 6.4 C-19 patients’ hospitalization

Many L-C19 sequelae have been observed after C-19 patients’ hospitalization. At 6-months after hospitalization, 90% of patients presented anxiety, depression, and sleep disorders (173), and the most prevalent symptoms reported at 12 months after discharge were anxiety, dyspnea, and fatigue (174). Evidence shows that individuals hospitalized for C-19 were more predisposed to develop anxiety than those hospitalized for other causes. Furthermore, hospitalized older patients were more predisposed to memory loss or confusion (175). The symptoms attributed to hospitalization are strongly correlated with other factors or comorbidities. For example, women with obesity were more predisposed to severe L-C19 at one year after discharging due to hormonal, pro-inflammatory and metabolic state (176).

When comparing hospitalized with non-hospitalized patients, *O’Mahoney et al.* reported that the five most common symptoms of hospitalized patients were fatigue (28.4%), pain/discomfort (27.9%), sleep difficulties (23.5%), breathlessness (22.6%), and limited regular activity (22.3%). Other symptoms included changes in lung structure/function, ground glass opacification, fibrotic changes, and reticular patterns. The non-hospitalized patients presented similar symptoms, however, with less incidence: fatigue (34.8%), breathless (20.4%), muscle pain/myalgia (17.0%), sleep disorders (15.3%), and loss of smell (12.7%) (177). Other symptoms observed in non-hospitalized patients were pneumonia, chest pain, palpitation, diabetes mellitus, and alopecia (178).

### 6.5 Reinfection

Reinfection was defined as the presence of new C-19 symptoms that occur more than 90 days after the previous diagnosis of confirmed SARS-CoV-2 infection (179). The severity of reinfection depends on the severity of the initial episode and is strongly correlated with genetic factors particularly related to the innate immune response and pathogenicity of the specific variant. Reinfections increased the development of L-C19, but were less identified in mild or asymptomatic patients, children and adolescents (180, 181). The most vulnerable to reinfections were healthcare workers who have been predisposed to C-19 infection since the beginning of the pandemic. The most frequent reported symptoms of L-C19 were asthenia (14.2%), cough (8.9%), myalgia (3.0%), dyspnea (2.0%), anosmia (1.8%), concentration deficit (1.5%), and headache (1.4%). When there were two symptoms, fatigue was always one of them. Other symptoms were related to gastroenteric and pulmonary dysfunctions. The most persistent symptoms were those related to neurological (23.3%) and psychological symptoms (18.2%), even after 61 days since the first negative swab (182). Reinfections of healthcare workers led to mild L-C19 symptoms, since most were younger than 65 (179).

### 6.6 SARS-CoV-2 strain/variant

In addition to host factors such as immunity status, comorbidities, vaccination status etc., Long COVID is also influenced by the virus, including strain/variant (180). Therefore, it is important to distinguish between long COVID infections with the wild type virus and subsequent variants. For instance, a significant decrease in cardiac symptoms, such as chest pain and palpitations were observed during Variant of Concern (VOC) infections periods, compared with wild-type virus infections. This decrease was sufficiently robust in adjusted statistical models, suggesting that this change occurred as a result of different infecting variants. However, the broad spectrum of symptoms belonging to either musculoskeletal or cardiorespiratory symptom clusters was similar in both wild-type and VOC infections, though more studies with higher number of observations/patients are needed to confirm the effects (183). Other studies reported differences in clinical symptoms between viral variants. For instance, most severe symptoms were observed in wild-type, during early waves of COVID, when most symptoms affected upper respiratory and central neurological systems, while anosmia, abdominal symptoms and a vast array of other symptoms were observed in alpha and delta variant infections (184). Similar findings point to the fact that wildtype SARS-CoV-2 infections had the highest post COVID condition (PCC) risks, with 6.44 times higher than subsequent variants. It has been suggested that previous natural infections, rather than vaccination, reduced the PCC risk, implying that natural immunity does not wane and is efficient for protection, including post COVID conditions. Another explanation is the possibility that individuals who did not develop PCC after the first infection, have specific traits that lower the risk of developing the condition after subsequent infections (185). The later variants, such as Omicron or Epsilon, were associated with reduced risk of developing long COVID (186). The fact that initial infections, due to more virulent strains such as the original Wuhan strain and the Alpha variant, are associated with a higher risk of long COVID may be due to an erratic and overwhelmed immune response following SARS-CoV-2. Thus, the possibility of developing severe symptoms is more likely. Also, the immunity status developed due to previous infections could play a role. However, factors such as vaccination, subsequent infection waves or social influences, such as post-traumatic stress disorder, physical inactivity, and lack of exercise during lockdowns may have confounded reports of emotional or cognitive symptoms, which may not be a true reflection of post-C OVID conditions (187). Notwithstanding, there is a general consensus that large cohort studies are needed to better discriminate between the factors leading to post COVID occurrence.

### 6.7 Vaccination

Several studies have shown that more than half of the vaccinated individuals have fewer symptoms than those who were not immunized by vaccination (188, 189). The most notable differences were observed in fatigue, brain fog, myalgia, and shortness of breath. It seems that these differences were strongly correlated with vaccine type. In their study on 812 L-C19 vaccinated participants, *Strain et al.* showed that the average symptoms score was greatly improved after vaccination with Astra Zeneca/Oxford vaccine being more efficient only for the fever. The mRNA Moderna vaccine was the best in reducing fatigue, brain-fog, myalgia, gastro-intestinal symptoms and autonomic dysfunction of L-C19. The average symptoms improvement score was 22.6% after Astra Zeneca/Oxford vaccine, 24.4% after the Pfizer/BioNTech vaccine, and 31% after Moderna vaccine. The authors concluded that mRNA Moderna and Pfizer vaccines presented more advantages compared to the modified adenoviral vector vaccine from Astra Zeneca (190). A similar pattern was observed by *Notarte et al.* in their meta-analysis of the effect of vaccination against L-C10 symptomatology. In addition, the latter study reported that two doses of vaccine were more effective to reduce the risk of L-C19 than a single dose, which was in line with other studies (191, 192). The time of vaccination was also very important, suggesting that individuals who were vaccinated a month before getting infected had reduced risk of experiencing L-C19 symptoms (193). By contrast, *Ayoubkhani et al.* showed that L-C19 symptoms decreased after the second dose of vaccine, regardless of the type and the time of immunization. If after the first dose, the loss of smell and taste, and trouble sleeping were reduced, after the second dose, the main decrease were observed for fatigue and headache (194). The incidence rate for L-C19 was lowest in individuals vaccinated with the third dose and the symptoms were more persistent in infection with delta and omicron SARS-CoV-2 variant (195–197). Norway national health care programs suggested that vaccination should be considered especially for non-hospitalized patients who experienced mild C-19 because they did not completely recover even eight months after infection (198). Similar results have been reported by *Yelin et al.* who concluded that almost 60% of individuals with mild or moderate C-19 experienced L-C19 symptoms, with difficulties in returning to the previous way of life (199). Irrespective of the type of vaccine and number of doses, the immunized patients presented an overall improvement in L-C19 symptoms, especially fatigue, breathless or insomnia, quality of life and wellbeing (200, 201).

### 6.6 Lifestyle

The lifestyle before the acute C-19 phase is also important in the development of symptoms associated with L-C19. For example, L-C19 symptoms were common in smokers and in individuals who worked from home and used car or public transportation instead of walking or cycling (202). This was also confirmed by *Wright et al.* who showed that physical activity improved mental health and improved quality of life, although it increased fatigue. These results are in line with NICE recommendations that limits graded exercise therapy due to the possibility of symptoms worsening (203). Air pollution has also been associated with L-C19 symptoms’ persistence with patients’ exposure to pollutants being strongly correlated with increased levels of inflammatory cytokines and proteins. Furthermore, the virulence was higher in air polluted areas, with adverse effects on respiratory diseases (204).

## 7 Management and countries approach to long COVID-19 management and treatment

During the C-19 pandemic, many governments have established strategies and took mitigation actions to reduce and stop the pandemic such as quarantine, social isolation, or confinement. This led to reduction in physical activity, with a negative impact on nutrition behavior and resultant composition of gut microbiota, with major implications in the severity of C19 (3). The burden of COVID as well as available treatments, resources and research have revealed significant disparities between affected communities with different income. However, the evidence on developing long COVID based on income and socio demographic factors is scarce. Determining the number of patients from those infected who later experience persistent symptoms is extremely difficult although it is estimated that between 10% and 45% experience long COVID (205). Therefore, there is an urgent need to identify and address global inequities in access to testing, surveillance, vaccinations and treatment. Very few studies have been conducted in low-income countries, such as Africa. The inadequate medical system, the challenge of following-up patients with L-C19, the lack of multidisciplinary services for patient rehabilitation, insufficient grants for research, and even inadequate funds for treatments and vaccines are significant problems that require comprehensive strategies (206). In general, individuals from middle and low-income countries who have developed L-C19 presented similar symptoms. For example, in African populations, fatigue was the most common symptom (26-56%), followed by confusion or lack of concentration (12-68%) and dyspnea (12-38%). Risk factors for L-C19 development were female sex, comorbidities, such as chronic illness, obesity, hypertension, hyperlipidemia, diabetes mellitus, and cardiovascular diseases, or lack of vaccines (207). Patients from low incomes countries have limited access to treatments, vaccines, and proper nutrition thus ensuring access for all individuals to quality medical services, rehabilitation, and disease management is critical (208).

As early as March 2021, the WHO Regional Office for Europe developed protocols and guidelines for long COVID with several countries following suit on developing their own strategies. For example, Denmark was the first country that published national guidelines for managing the effects of L-C19 (November 2020, revised March 2021). On February 2021, France came up with its own guidelines, focusing on treatment and prevention of L-C19. This included rehabilitation centers for patients with severe problems and physiotherapy delivered through outpatient units for those with less severe symptoms. The Netherlands employed online strategies for patients and their representatives. For instance, “Long-form Corona Line” is a platform were L-C19 patients share their experiences with other patients and access medical experts; “Support around Corona” is a platform specialized in psychotraumatology, which offers support for mental health issues that is available to the public and health professionals. On the other hand, Georgia’s rehabilitation program focused on L-C19 patients with pulmonary symptoms, who received long-term treatment in centers (more than 21 days). Other measures included patient education, training and support for physicians. Poland was the first country that applied WHO’s recommendations regarding physiotherapy for L-C19 patients. Portugal developed a national guideline for L-C19 management, with focus on multidisciplinary management in primary care. In Italy, the National Institute of Health and academic centers developed and implemented multidisciplinary approaches that involved clinical, psychological and neurological factors. Austria developed multi-centers that followed-up patients with severe L-C19 symptoms in their first, third and sixth month after discharge. In Kazakhstan, the rehabilitation of L-C19 patients is provided mainly in hospitals, particularly for those with severe manifestations of L-C19 symptoms that include immune deficiency conditions, cognitive disorders, progression of atherosclerosis and exacerbation of hypertension (209). **Table 3** depicts several examples of global good practices that may be used as models for recognizing, diagnosing, and managing L-C19.

**Table 3:** Measures taken by countries in the management of L-C19.

Country/Group of countries	Actions	Reference
Austria	*Long Covid Europe*, new network of patients’ association through social media websites.	(210)
France	PET metabolic pattern recommendations to simplify the visual interpretation of neurological L-C19 signs in clinical routine.	(211)
	Guidelines published by French National Authority for Health for L-C19 patients follow-up.	(210)
Germany	Guidelines and recommendations by Robert Koch Institute, that advise the German Ministry of Health; C19 patients’ financial or in-kind support. Higher salary and extra remunerations for care workers; health care workers catering support from the Bavarian Minister for Health and Care and the Bavarian Minister of Finance.	(212)
	German PAC-19QoL, an instrument for establishing quality of life and daily clinical practices for patients with L-C19 syndrome.	(213)
	Post-C19 syndrome validated scoring system with benefits in preventing care medicine, clinical management of L-C19 disorders, and prioritization of healthcare services.	(214)
Italy	Emphasize patient follow-up, specific clinical needs, and individualized care plans.	(215)
	Address communication gaps between citizens’ needs and public government measures.	(216, 217)
Denmark Finland Iceland Norway Sweden	Outpatients’ clinics for patients with L-C19 symptoms. Follow-up guidelines implemented by the National Board of Health and Welfare.	(210)
	Strengthening Nordic cooperation to manage all C19-related issues.	(218)
	Apply strategies developed for C-19 pandemic to support companies to reduce the risks of rising unemployment; benefits for families with children and at least one unemployed member of family; extra-funding for social assistants.	(219)
	National and regional guidelines, with regulations specific for every country.	(220–222)
Spain	Clinical practice guidelines for L-C19 patients non-hospitalized in acute phase, whose follow-up and management are under hospital outpatient department or primary care centers’ responsibility.	(223)
	Spanish EuroQol−5D−5L validated questionnaire applied as quality-of-life measurement instrument.	(224)
	Special follow-up for patients with previous lung diseases and tobacco consumption, as well and children and women. Access to active community resources: green spaces, local facilities, physical and cultural activities.	(225)
UK	Telemedicine and telerehabilitation with multidisciplinary approaches and trained teams. Multicenter for post hospitalization C19 (PHOSP-COVID), with personalized proactive approach and holistic critical care for long-term follow-up of L-C19 patients. Personalized L-C19 clinical programs based on previous hospitalization of C-19 patients, non-hospitalized patients with persistent symptoms, or existing diseases complicated by C-19.	(226)
	L-C19 patients self-management through home monitoring: symptom trackers assessment, pulse oximeters or thermometers.	(227)
	Budget allocation for local health boards to help L-C19 patients and for development of L-C19 clinics.	(204)
	Budget allocated to establish the epidemiology, symptomatology, phenotyping, risk factor and risk category, as well as novel treatments and strategies to manage L-C19; multidisciplinary team collaboration with patients for targeted therapeutic approaches development.	(228)
USA	Investments in treatments and therapeutic approaches for targeted therapies.	(229)
	*RECOVER* initiative of $1.15 billion nationwide research program; Office of Long COVID Research and Practice and launch Long COVID clinical trials.	(230)
	National clinical trials to develop treatments for L-C19, that include drugs, biologics, medical devices, and other therapies.	(231)

## 8 Therapeutical approaches for long COVID-19

Prospective interventions studies on L-C19 include rehabilitation programs (on-site or online) for cognitive disorders, targeted drug therapies (specific drugs or microbiota modulating drugs), metabolic modulators, immunomodulatory therapies, antifibrotic and anticoagulation therapies (232, 233). Supplementation with vitamins such as B2, E, C and antioxidants have also been recommended and may represent a potential therapeutic strategy for neurorehabilitation. Evidenced obtained thus far concluded that some pharmaceutical treatments such as antidepressants have no effect, and non-pharmaceutical procedures, such as cognitive-behavior therapy, graded-exercise-related therapies, rehabilitation, or acupuncture showed inconsistent results. Since L-C19 affects multi organ systems, a multidisciplinary approach is required for its management and treatment. Hence, for a better understanding of the pathology and therapeutic approaches, the early initiation of treatment is crucial, and the strategies must be the result of teamwork with different expertise, such as neurologists, psychiatrists, psychologists, physiotherapists, occupational therapists, geriatric, respiratory, cardiovascular, endocrine, renal, hematologic, or autoimmune disease specialists (174, 234, 235). Other potential therapeutic approaches for L-C19 are presented in **Table 4**.

**Table 4:** Alternative therapeutical approaches to L-C19 sequelae.

Alternative treatment	Results	References
Cardiorespiratory and musculoskeletal fitness	After 8 weeks intervention (3 session per week), muscle strength increased from 16% to 33%, and peak aerobic exercise capacity increased by 15%.	(236)
6-min walk distance exercises	During 6 weeks of testing, L-C19 patients presented increased pulmonary function, and improvements in dyspnea, fatigue, and quality of life.	(237)
Sodium–glucose cotransporter 2 inhibitors and glucagon-like peptide 1 receptor agonists	Cardiovascular and renal benefits, improved metabolic function.	(160)
Systemic corticosteroids	After 6-8 weeks treatment, fatigue decreased by 41%, breathlessness by 46%, and cough by77%.	(238)
Probiotics and short-chain fatty acids (SCFA)	Restored gut microbiota and decreased GI disorders; improved mental health due to gut- brain axis modulation; SCFA maintained intact blood-brain barrier, downregulated microglial activation and reduced inflammation.	(239, 240)
L-Arginine and liposomal Vitamin C	1.66 g of L-arginine and 500 mg liposomal Vitamin C for 28 days improved endothelial function, walking performance, reduced fatigue, and normalized pulmonary arterial hypertension.	(241)
High-dose intravenous vitamin C	100-300 mg/kg body weight for 7 days improved oxygenation and reduced inflammation; ameliorated fatigue and associated symptoms such as sleep disorders, depression or pain.	(242)
Vitamins (B group, C, D, E) and minerals (magnesium, zinc, selenium)	Preventive effect on the development of autoimmunity, on alveolar damage, endothelial dysfunction, infection fervency reduction, balancing gut microbiota and coagulation.	(243)
Pan-caspase inhibition	Improved fatigue, dyspnea, cough, joint pain, anosmia, rheumatic fever.	(244)
Acupuncture	After 6 sessions, brain fog improved and joint pain reduced; blood stagnation reduced.	(245)
Rosemary Carnosic Acid	Neuroprotective and anti-inflammatory effects (limiting IL-1 β production by inhibition of NLRP3 inflammasome activation)	(246)

Irrespective of the proposed therapeutic method, it is essential that individuals suffering from L-C19 be included in rehabilitation programs to identify physical, emotional, cognitive and social treatable traits. The issue may be even more pressing for those who, after 6 months of L-C19, not only continue to experience significant symptoms but also to identify new ones (247).

Pharmacological treatment approaches include treatment with β-blockers, fludrocortisone, midodrine, increase in salt and fluid intake, intravenously administered salt, compression stockings for postural orthostatic tachycardia syndrome (POTS) and even for myalgic encephalomyelitis or chronic fatigue syndrome treatment (248). Administration of intravenous immunoglobulin can be considered for immune dysfunction and a new drug, BC007 who neutralizes G protein-coupled receptor autoantibodies has shown promising results for autoimmunity that occur in L-C19 (249). Administration of nirmatrelvir-ritonavir has been used for patients with persistent SARS COV-2, since some studies suggest that the persistence of SARS-CoV-2 in tissues and especially in the intestinal microbiome and virome may be involved in the pathogenesis of L-C19 (250). The use of valacyclovir, valganciclovir, famciclovir for reactivation of infections with Epstein–Barr virus, cytomegalovirus and varicella zoster virus may help prevent the neurological manifestations of L-C19, because reactivated herpesviruses are also associated with ME/CFS (251). Anticoagulants can prevent abnormal clotting and can be useful in L-C19, with some studies recommending even triple anticoagulant therapy (100, 252). Apheresis can also help to remove microvascular blood clotting and has been shown to reduce autoantibodies in ME/CFS, but high costs limit its use (253). Finally, Coenzyme Q10 and d-ribose have been shown to have a beneficial role in asthenia and neurological symptoms found in MEC/CFS (254).

## 9 Imaging investigations in long COVID-19

Imaging studies have been used to evaluate lung diseases both in detecting the degree of damage in the acute phase and in the evaluation of post-COVID sequelae. Computer tomography (CT) scans are the standard method for accurately estimating the lung area affected by the infection. Imaging analyses have been less used in investigating the long-term effects of C-19. L-C19 causes damage to other organs such as the kidneys, heart, and brain, thus using MRI examinations of COVID patients showed multiorgan abnormalities in 61% of patients vs. 14% in non-infected controls that were seen in the lungs, kidneys, and brain. At five months after discharge, a significant number of patients had lung damage in excess of 5%. These findings underscore the need for imaging surveillance of patients in addition to blood tests or detailed medical history evaluation (255).

## 10 Current and future research opportunities

L-C19 management involves physical rehabilitation which should begin in the acute phase because early mobilization could reduce hospitalization and functional outcomes; management of pre-existing comorbidities that can avoid clinical deterioration, readmission of patients and avoid other pathologies and symptoms development; mental health support, or social services support. The management of patients include patient-reported outcomes for long-term follow-up care and for early detection of possible adverse events and the use of digital technologies, such as telemedicine - videoconferences for patients’ follow-up and reinfection risk elimination, digital therapeutics approach, especially for non-pharmaceutical interventions (e.g., breathing exercises), electronic control of body parameters such as temperature, oxygen saturation, blood pressure, that can be measured with wearable devices and the data can be analyzed with artificial intelligence (256, 257). In their Delphi study, *Nurek et al.* suggested that recommendations for the recognition, diagnosis, and management of L-C19 should meet several criteria related to i) clinic organization (cross-specialty doctors, multi-specialized medical team, individualized investigations, patients’ equitability regardless of the comorbid mental health difficulties), ii) diagnosis of disorders (specific guidelines, early-stage symptoms’ identification), and iii) management (patients’ follow-up and support, encouraging the report of new signs and symptoms) (258). Healthcare and research workers training and education, public communication campaigns and research funding should be one of the main goals of L-C19 management (259).

Current priorities for research should include: i) determining the L-C19 sequelae and symptoms in early stage of development; ii) identifying the pathophysiological mechanisms that contribute to L-C19 development; iii) evaluating the long-term impact for patients with other pre-existing co-morbidities, hospitalized or with ICU interventions in acute C-19 phase; iv) establishing strategies and regulations for L-C-19 recovery; v) identifying therapeutic solutions, innovations in novel fast-acting therapies and drugs, vi) evaluating the role of vaccination; vii) understanding the impact of L-C19 on patients’ daily life, management and healthcare costs (260). These strategies should be corroborated with emerging viral mutations that have been reported (**Table 5**).

**Table 5:** SARS-CoV-2 variant classifications [on September 1^st^, 2023, according to (261, 262)].

WHO Label	Pango Lineage	VBM *
Mu	B.1.621, B.1.621.1	September 21, 2021
Zeta	P.2	September 21, 2021
Omicron	B.1.1.529	November 26, 2021
	BA.2.74, XBB.1.5; XBB.1.9.1; CH.1.1; XBB.1.16; XBB.2.3; XBB.1.9.2; BA.2.86	September 1, 2023
N/A	B.1.617.3	September 21, 2021
Kappa	B.1.617.1	September 21, 2021
Iota	B.1.526	September 21, 2021
Eta	B.1.525	September 21, 2021
Epsilon	B.1.427; B.1.429	September 21, 2021
Delta	B.1.617.2	September 21, 2021
Gamma	P.1	September 21, 2021
Beta	B.1.351	September 21, 2021
Alpha	B.1.1.7	September 21, 2021
N/A	Variants containing the F456L spike mutations	September 1, 2023

*VMM, variants being monitored.

Whether the newly emerging variants are likely to cause more serious effects is not yet clear, predicting their long-term effects is also challenging. Thus, devising novel strategies such as examination of SARS-CoV-2 spike protein epistatic networks may be useful in forecasting viral haplotypes with high transmissibility (263). Notwithstanding, the COVID-19 pandemic taught us that, uncertainty is specific to such viruses that undergo multiple mutations within a short period of time, thus it is incumbent on the research community to decipher the mechanisms of action and to predict as best as possible the likely consequences (264).

## Author contributions

RG: Conceptualization, Data curation, Investigation, Methodology, Writing – original draft. IS: Data curation, Writing – original draft. AL: Data curation, Writing – original draft. OS: Data curation, Writing – original draft. RF: Conceptualization, Writing – original draft. AC-B: Data curation, Writing – original draft. MD: Funding acquisition, Investigation, Project administration, Resources, Supervision, Writing – review & editing. SM: Data curation, Funding acquisition, Project administration, Writing – review & editing. MC: Data curation, Funding acquisition, Investigation, Project administration, Resources, Supervision, Writing – review & editing.
