# Supplementary material for: The knowns and unknowns of long COVID-19: from mechanisms to therapeutical approaches
Source: Front Immunol. 2024 Mar 4;15:1344086. doi: 10.3389/fimmu.2024.1344086 (PMC10944866; doi:10.3389/fimmu.2024.1344086)
Supplement: Supplementary file 1 [file DataSheet_1.pdf]

Supplementary material

Appendix 1.

Table 1.1. L-C19 symptoms and their substrate and treatment

Symptoms	Modifications	Treatment	References
PSYCHIATRIC<br><br>bipolar disorder<br>post-traumatic stress disorder<br>psychiatric disorder<br>substance use disorder	Gliosis in ventral striatum and dorsal putamen<br>Neurotransmitter depletion<br>Synaptic pruning<br>Reduction in gray matter thickness and global brain size, larger in older ages	Anxiety disorders<br>Reduce psychiatric medication<br>Good sleep	[271-275]
Neurologic<br>Fatigue	Low level of activity<br>Disrupted sleep wake pattern	Remediation through training	[276-278]
Cognitive impairment	Brain fog<br>Problems in attention, executive functions, language, memory	Compensatory techniques	[279,280]

Table 1.2. Correlation between symptoms and laboratory test results in L-C19

Symptoms	Laboratory test markers	References
Psychiatric	CRP ↑<br>Interleukin 6 ↑<br>Lymphocyte count ↓	[281-283]
Neurologic injury	D dimers ↑<br>Ferritin ↑<br>TNF α<br>CRP ↑<br>Albumin ↑<br>Interleukin 6<br>Proinflammatory cytokines ↑<br>Interleukin 1 β ↑<br>Thrombophilia<br>Complement ↑<br>Cerebrospinal fluid (CSF) abnormalities<br>- CSF protein ↑<br>- Abnormal oligoclonal binding<br>- Intrathecal IgG antibody production<br>- Auto antibodies to neural tissue and prothrombotic antibodies	[284-287]
Pulmonary fibrosis	IL-6 ↑	[288]

	CRP ↑<br>TGF β ↑	
Subacute thyroiditis	ESR ↑<br>CRP ↑<br>WBC ↑<br>FT4↑<br>FT3↑<br>TSH- not detectable<br>ATPO negative	[289]

Abbreviations: CRP, C reactive protein, TGF β, Transforming growth factor, Il-6, Interleukin 6, ESR, Erythrocyte Sedimentation Rate, WBC, White Blood Count, ATPO, Anti-tyroidperoxidaze.
